# Inhibition of Adrenergic Receptors Reduces Genital HSV-1 Clinical Recurrences in Guinea Pigs

**DOI:** 10.3390/v18101081

**Published:** 2026-10-01

**Authors:** Jillian C. Green, Greyson A. Moore, Matthew D. Irwin, Jonathan D. Joyce, Andrea S. Bertke

**Affiliations:** 1 Biomedical and Veterinary Sciences, Virginia-Maryland College of Veterinary Medicine, Virginia Tech, Blacksburg, VA 24061, USA; greenji@vt.edu (J.C.G.); gamoore@vt.edu (G.A.M.); mdirwin@vt.edu (M.D.I.); 2 Center for Emerging, Zoonotic, and Arthropod-Borne Pathogens, Virginia Tech, Blacksburg, VA 24061, USA; jjoyce84@vt.edu; 3 Department of Population Health Sciences, Virginia-Maryland College of Veterinary Medicine, Virginia Tech, Blacksburg, VA 24061, USA

**Keywords:** herpes simplex virus, HSV-1, recurrences, epinephrine, adrenergic, adrenergic receptors, peripheral nervous system, sympathetic nervous system, propranolol, beta blocker, beta adrenergic receptor

## Abstract

Herpes simplex virus 1 (HSV-1) establishes latency in both sensory and sympathetic neurons. Stress is strongly correlated with HSV-1 reactivation. The acute stress hormone epinephrine initiates HSV-1 reactivation in vitro upon activating at least two adrenergic receptors (ARs), including α2, β1, and/or β2-AR. To determine if blocking these receptors could reduce HSV-1 recurrences, we infected guinea pigs vaginally with HSV-1 (17+) and, after latency was established, treated them daily with atipamezole (specific α2-AR inhibitor), propranolol (nonspecific β-AR inhibitor), or atipamezole/propranolol combined. Clinical recurrences decreased in the treatment groups compared to untreated: atipamezole (29.3%, *p* = 0.123), propranolol (43.6%, *p* = 0.021), and atipamezole/propranolol (39.1%, *p* = 0.021). No significant differences were observed between groups in HSV-1 viral load in pelvic ganglia, or in transcription of the latency-associated transcript (LAT) in guinea pigs that were asymptomatic when euthanized, suggesting AR inhibitors did not affect latency. In guinea pigs euthanized during recurrences, viral DNA was similar between groups in sensory ganglia but reduced in sympathetic ganglia in treatment groups compared to untreated, suggesting that reactivation still occurred in sensory but was limited in sympathetic neurons in AR inhibitor-treated animals. The use of adrenergic beta blockers may be a useful therapeutic to reduce HSV-1 clinical recurrences.

## 1. Introduction

Herpes simplex virus 1 (HSV-1) is a DNA virus that causes lifelong infection, with the potential to cause repeated episodes of recurrent disease. An estimated 3.8 billion adults under age 50 worldwide currently have HSV-1 [1]. Although historically, HSV-1 was acquired in childhood as oral herpes, the virus is increasingly diagnosed as a genital infection in adults [1]. In 2020, an estimated 376.2 million individuals had existing HSV-1 genital infection and 16.8 million new HSV-1 infections were diagnosed globally [1]. In addition to causing oral and genital lesions, HSV-1 can also reach the brain, where it can cause life-threatening encephalitis in about 2.2 people per million globally each year [2], and has been implicated in the development of Alzheimer’s disease [3,4,5].

HSV-1 is transmitted through bodily fluids, including saliva and genital secretions, as well as through direct contact with herpetic lesions [6]. During primary infection, the virus replicates in the epithelium, causing a vesicular skin lesion at or near the site of infection, and gains access to sensory and autonomic nerve endings innervating these areas. Following retrograde transport to the neuronal cell nuclei, HSV-1 establishes latency, during which no new infectious viral particles are produced and the viral genome remains in a latent state for the life of the host, although the virus can periodically reactivate to cause recurrent disease [6].

Various stimuli are known to cause reactivation of HSV-1 from latency, resulting in the production of new infectious virions [6]. These new virions travel anterograde from the neuronal cell body through the axons to the innervated epithelium, resulting in new herpetic lesions and viral shedding. Known stimuli for reactivation include nerve damage [7,8], immune system dysregulation [9], hormone fluctuations [10,11,12,13], and stress [10,12,13,14,15,16,17,18,19,20], although many recurrences occur spontaneously without an identifiable trigger [21]. Both acute and chronic stress have been implicated in inducing HSV-1 reactivation. Chronic stress, characterized by prolonged elevation of cortisol, causes immune suppression [22] but also directly reactivates latent HSV-1 in cultured neurons [10]. The acute stress response includes a hormonal surge of epinephrine, activated by the sympathetic nervous system and hypothalamic–pituitary–adrenal (HPA) axis [23].

Epinephrine is a catecholaminergic stress hormone that modulates the “fight or flight” response [24]. During an acute stress event, epinephrine is released from the adrenal glands into circulation, where it can bind to adrenergic receptors (ARs) found throughout various body systems. ARs are membrane-bound G protein-coupled receptors (GPCRs) that can activate distinct intracellular signaling cascades, depending on the receptor subtype and the alpha subunit of the associated tripartite G protein. Upon activation, the G alpha (Gα) subunit dissociates from the beta and gamma (Gβγ) complex, initiating signaling through pathways such as the cyclic AMP (cAMP) and phosphatidylinositol cascades. α2-AR activates Gαi/o, while β1-AR activates Gαs, and β2-AR activates Gαs or Gαi/o. Activation of these pathways mediates physiological responses associated with an acute stress response, including increased blood pressure, accelerated heart rate, and vasoconstriction [25,26]. ARs are expressed throughout the body on many cell types, including both sensory and autonomic neurons [27]. We previously demonstrated that epinephrine treatment results in increased HSV-1 DNA viral load and infectious virus during a productive infection [18] and induces reactivation from latency in primary sympathetic neurons but not sensory or parasympathetic neurons [10]. In primary mature cultured neurons, reactivation of the virus required activation of at least two different ARs, including any combination of α2, β1 and β2 [10].

In this study, our goal was to determine if blocking α2, -β1 and -β2 ARs could prevent or reduce HSV-1 recurrences following HSV-1 genital infection in guinea pigs. Following infection with HSV and clearance of acute disease symptoms, guinea pigs experience spontaneous recurrences without exogenous induction stimuli. Furthermore, recurrence frequency increases in response to stress. Since we identified α2, β1 and β2 ARs as critical for reactivation of HSV-1 in primary sympathetic neuronal cultures, we hypothesized that blocking these receptors in vivo would reduce recurrences originating from sympathetic neurons in the sacral sympathetic ganglion and the presynaptic sympathetic center in the spinal cord.

## 2. Materials and Methods

### 2.1. Cells and Viruses

Stocks of HSV-1 strain 17+ were propagated in Vero76 cells (CRL-1587, ATCC, Manassas, VA, USA) in Dulbecco’s Modified Eagle’s medium (DMEM, Millipore Sigma, Burlington, MA, USA) with 5% fetal bovine serum (FBS, Millipore Sigma, Burlington, MA, USA) and 100 U/mL penicillin G and streptomycin (Millipore Sigma, Burlington, MA, USA). Standard plaque assays were performed with Vero76 cells to quantify viral titers.

### 2.2. Guinea Pig Inoculation, Clinical Assessment, and Tissue Collection

Three-week-old female Hartley guinea pigs (Hilltop Laboratories, Scottdale, PA, USA) were anesthetized with ketamine and xylazine and intravaginally infected with HSV-1 (strain 17+, 2 × 10^5^ PFU, n = 64) or mock infected with phosphate buffered saline (PBS, n = 9) (Millipore Sigma, Burlington, MA, USA), using the well-established model of HSV infection in guinea pigs [28,29,30,31,32]. The 64 infected guinea pigs were randomly assigned to four treatment groups, including untreated (n = 16), atipamezole (n = 16), propranolol (n = 16), and atipamezole/propranolol (n = 16). Treatments did not begin until 15 days post-inoculation (dpi). One guinea pig in the untreated group died during acute disease and was removed from the study, leaving the untreated group with 15 animals (n = 15). During the period of acute disease (1–14 dpi), genital lesion severity was measured on an established four-point scale: 0 = no symptoms, 1 = redness and/or swelling, 2 = 1–2 vesicular lesions, 3 = 3–5 vesicular lesions, and 4 = 6+ lesions or a coalescence of lesions. Weight and clinical scores were documented daily throughout the study period (0–60 dpi). Beginning 15 dpi at the conclusion of the acute disease period, guinea pigs were administered daily intraperitoneal (IP) injections of 1.0 mg/kg atipamezole to block the α2-AR (Millipore Sigma, Burlington, MA, USA), 0.5 mg/kg propranolol to block β1-AR and β2-AR (Millipore Sigma, Burlington, MA, USA), or both atipamezole (1.0 mg/kg) and propranolol (0.5 mg/kg) to block all three adrenergic receptors (n = 16 per group) every 24 h, or were untreated (n = 15). Dosage was determined based on typical human dosing (40 mg/day for anxiety treatment), guided by and adjusted for recommended small animal dosing [33]. Recurrences were defined as presence of vesicular lesions, recorded on the first day of appearance, through the end of the study. Daily vaginal swabs were collected from 9 guinea pigs in each infected group and 6 guinea pigs in the uninfected group with pediatric FLOQSwabs (Copan Diagnostics, Murrieta, CA, USA) from 15–60 dpi and stored in DMEM at -80 °C for analysis of viral genomic shedding by qPCR. Nervous system tissues were collected from latent guinea pigs that had no visible recurrences at 60 dpi (untreated, n = 9; atipamezole, n = 9; propranolol, n = 9; atipamezole/propranolol, n = 9; uninfected n = 6) or recurrent guinea pigs during a clinical recurrence (untreated, n = 6; atipamezole, n = 7; propranolol, n = 7; atipamezole/propranolol, n = 7; uninfected, n = 3). Dorsal root ganglia (DRG), sacral sympathetic ganglia (SSG), and lumbar spinal cord (L-SC) were collected into TRI Reagent (Millipore Sigma, Burlington, MA, USA) for DNA and RNA extraction (DRG, n = 8 latent, n = 6 recurrent, n = 3 uninfected; SSG, n = 7 latent, n = 6 recurrent, n = 3 uninfected; and L-SC, n = 7 latent, n = 6 recurrent, n = 3 uninfected). Since one untreated guinea pig was removed from the study during acute disease, one less recurrent DRG, SSG, and L-SC was collected (n = 5). The remaining DRG, SSG, and L-SC were collected into paraformaldehyde (Electron Microscopy Sciences, Morgantown, PA, USA). Sample sizes were determined by power analysis, based on previous experimental results in similar studies.

### 2.3. Assessment of HSV-1 DNA Shedding in Vaginal Secretions

Vaginal swab samples were thawed on ice and vortexed. No DNA extraction was performed. Viral DNA was quantified by qPCR, using iTaq Universal Probe Supermix (Bio-Rad, Hercules, CA, USA) with 10 μM MgCl, primers and probe specific for the HSV-1 thymidine kinase (TK) gene, and 4 μL of swab sample per qPCR reaction. The QuantStudio 6 Pro real-time PCR system (Thermo-Fisher, Waltham, MA, USA) was used on the standard setting of 50 °C for 2 min, 95 °C for 10 min, 40 cycles of 95 °C for 15 s and 60 °C for 60 s. HSV-1 standards of known concentration were used to compare viral DNA quantities and values were reported per 1 mL [28].

### 2.4. DNA and RNA Extraction and TK-Specific qPCR and LAT-Specific RT-qPCR

Viral DNA and RNA were extracted from nervous system tissues stored in TRI Reagent (Thermo-Fisher, Waltham, MA, USA). All tissues were homogenized using a BeadBug3 (Benchmark Scientific, Sayreville, NJ, USA) for three cycles of 30 s each, incubated for 60 min on ice, then homogenized again 3 times for 30 s, and incubated again for 60 min on ice. DNA and RNA were extracted following manufacturer’s directions using the Direct-zol DNA/RNA Miniprep kit (Zymo, Tustin, CA, USA). Viral DNA was quantified by qPCR, using iTaq Universal Probes Supermix (Bio-Rad, Hercules, CA, USA) and primers and probe specific for the HSV-1 thymidine kinase (TK) gene. After reverse transcription of the RNA to cDNA using iScript Reverse Transcription Supermix for RT-qPCR (BioRad, Hercules, CA, USA) following the manufacturer’s protocol, transcription of the latency-associated transcript (LAT) was quantified using primers and probes specific for HSV-1 LAT in iTaq Universal Probe Supermix (Bio-Rad, Hercules, CA, USA). The QuantStudio 6 Pro real-time PCR system was used on the standard setting of 50 °C for 2 min and 95 °C for 10 min, followed by 40 cycles of 95 °C for 15 s and 60 °C for 60 s. Standards of known concentrations were used to compare unknown sample Ct values for quantification of transcripts. DNA and RNA copies are reported as fold change normalized to untreated guinea pig mean for latent guinea pig DNA, fold change normalized to untreated guinea pig mean per transcript/DNA for latent RNA, and as fold change of reactivating guinea pig DNA normalized to latent guinea pig mean DNA copy number [28].

### 2.5. Statistical Analysis

Statistical analyses were performed in JMP 19 (SAS Institute, Cary, NC, USA), using the following statistical tests: acute lesion severity, weight gain, and swab viral load used one-way ANOVA with Tukey’s post hoc test; tissue analyses used one-way ANOVA with Dunnet’s post hoc test with Untreated as the reference group.

## 3. Results

### 3.1. Clinical Disease

To determine if inhibition of adrenergic receptors (ARs) -α2, -β1, or -β2 can prevent or reduce genital HSV-1 recurrences, we infected female Hartley guinea pigs with HSV-1 (strain 17+) and treated them daily with atipamezole (α2 blocker, n = 16), propranolol (non-specific beta blocker, n = 16), or both atipamezole and propranolol (n = 16), starting after the acute disease period ended fifteen days post-inoculation (dpi), or left them untreated (n = 15). Daily, the guinea pigs were examined for genital lesions and weighed. Guinea pigs that remained in the study for 60 dpi were also swabbed to assess vaginal viral DNA shedding.

**Figure 1:**
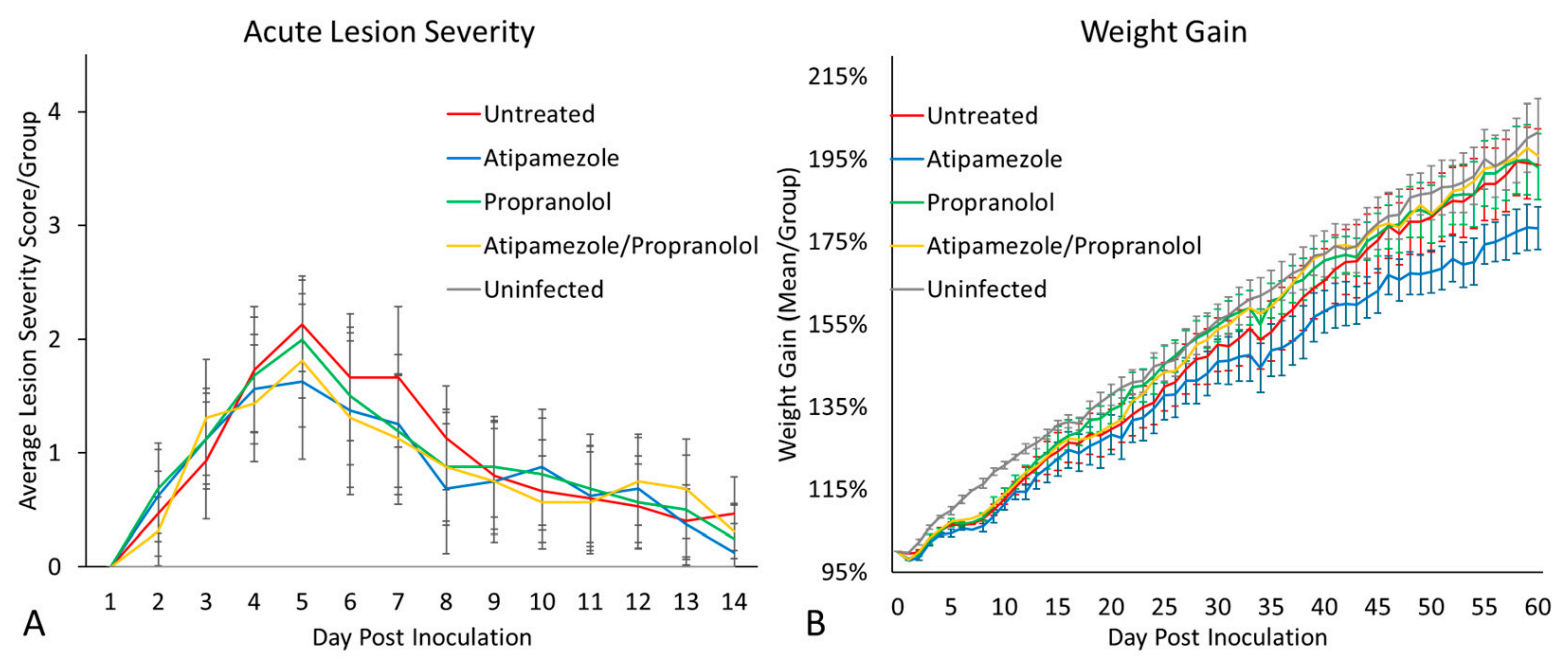
Acute clinical disease and weight gain. Guinea pigs were infected with HSV-1 (17+) and observed daily for lesions and weight. Treatment with adrenergic receptor inhibitors began 15 dpi, after the acute disease period concluded. (**A**) Acute lesion severity. Guinea pigs were scored daily for lesion severity during the acute disease period (1–14 days post-infection), using a four-point scoring system: 0 = no disease, 1 = redness/swelling, 2 = 1–2 vesicular lesions, 3 = 3–5 vesicular lesions, 4 = 6+ vesicular lesions or coalescence of lesions. No guinea pigs were treated during this period, but were designated to groups by treatment that commenced 15 dpi: untreated (n = 15), atipamezole (n = 16), propranolol (n = 16), atipamezole/propranolol (n = 16), uninfected (n = 9). No statistically significant differences in disease severity were observed between infected groups (one-way ANOVA with Tukey’s post hoc test), showing well-balanced groups. (**B**) Weight gain. Guinea pigs were weighed daily from 0 dpi to 60 dpi. Weight is reported as percent weight change mean per group, adjusted for number of guinea pigs each day. No statistically significant differences were found between groups (one-way ANOVA, Tukey’s post hoc test).

During the acute disease period (1–14 dpi), no significant differences in disease severity were noted between the groups, which was expected since no guinea pigs received drug treatment during this time (Figure 1A). These results verify that the groups were well-balanced in acute disease severity prior to treatment with adrenergic receptor blockers.

The weights for the uninfected guinea pigs increased from an average of 292.5 g baseline weight on the day of infection to 589 g by the end of the study at 60 dpi (201.6% increase), while those that were infected with HSV-1 but otherwise untreated increased 193.7% (Figure 1B). The weights for infected guinea pigs treated with propranolol (193.2% increase) or the combination of atipamezole/propranolol (195.9% increase) did not differ substantially from untreated infected guinea pigs or from uninfected guinea pigs. Guinea pigs treated with atipamezole alone, however, showed a slightly reduced rate of weight gain compared to the other groups (178.3% increase from baseline) (Figure 1B), perhaps due to transient hyperexcitability caused by daily treatment [34]. No statistically significant differences in weight gain were identified.

Beginning 15 dpi, guinea pigs were assessed daily for the presence of recurrent lesions. In the guinea pig genital model of infection, the end of the acute stage of disease is defined as 14 days post-infection. Guinea pigs infected with HSV experience spontaneous recurrences without exogenous stimuli, such as iontophoresis or hyperthermia used in the mouse or rabbit models [35]. However, the frequency of recurrences increases in response to stress, much like humans.

**Figure 2:**
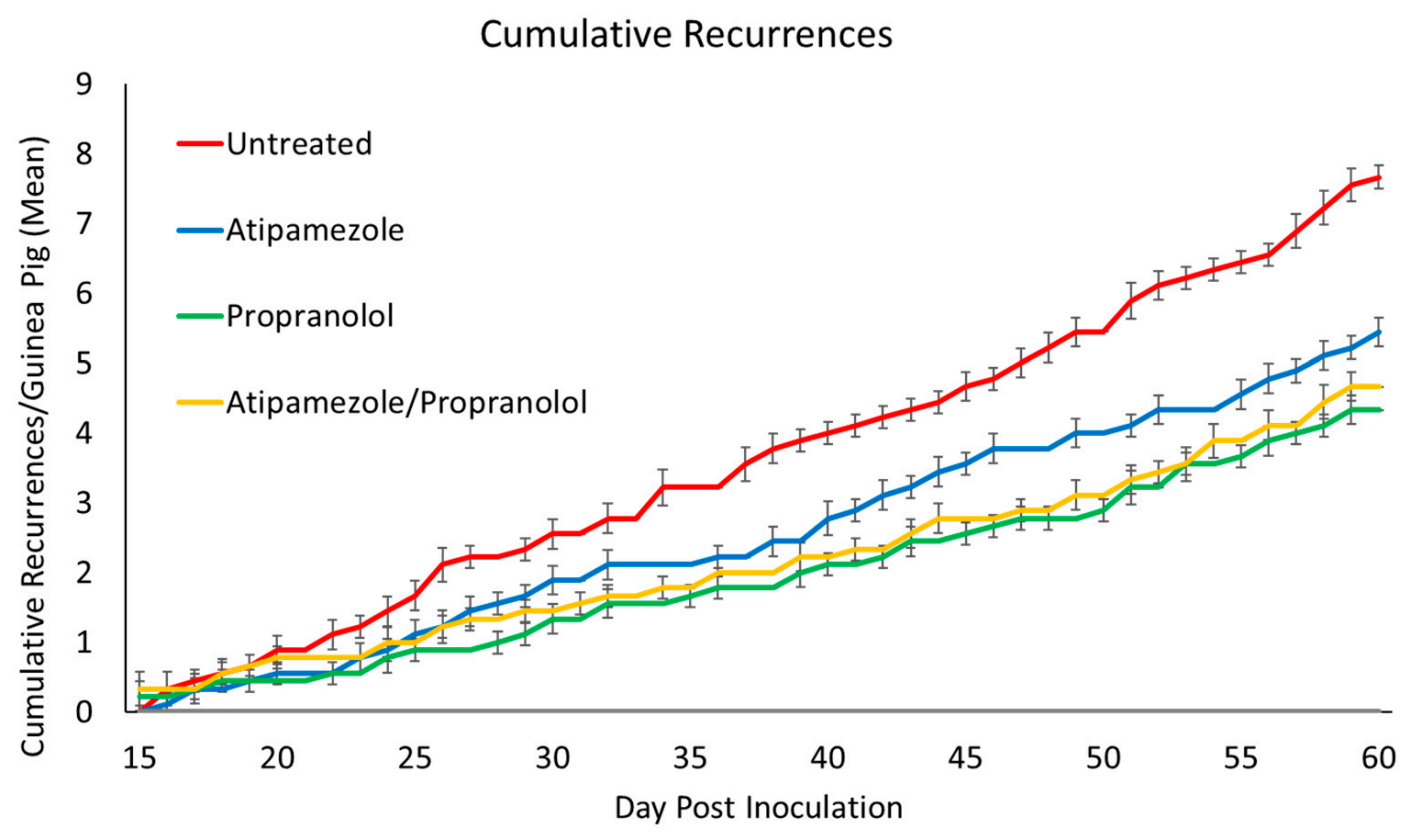
Cumulative recurrences. Guinea pigs were observed daily for genital HSV-1 recurrences, defined as the presence of a new vesicular lesion, recorded on the first day of observance. Compared to the HSV-1 infected untreated group (n = 9), guinea pigs treated with daily atipamezole (α2-AR blocker, n = 9) had a 29.26% reduction in recurrences (*p* = 0.1226). Those treated with daily propranolol (β blocker, n = 9) had a 43.5% reduction in recurrences (*p* = 0.018) and those treated daily with both atipamezole and propranolol (n = 9) had a 39.1% reduction in recurrences (*p* = 0.021). Uninfected controls had no recurrences (n = 6). One-way ANOVA with Dunnett’s post hoc test was used to determine significance. Guinea pigs in each group were randomly selected for euthanasia and tissue analysis during their first visible clinical recurrence, while others were allowed to continue until end of study (60 dpi); only guinea pigs that remained in the study through 60 dpi are included in cumulative recurrence data.

Some of the guinea pigs in each treatment group were randomly selected for euthanasia during their first recurrence after clearance of acute disease symptoms, to analyze tissues during these recurrences. Only guinea pigs that remained in the study until 60 dpi were included for calculation of cumulative recurrences (n = 9 per infected group, n = 6 uninfected) (Figure 2). Guinea pigs that were infected with HSV-1 but otherwise untreated experienced a cumulative average of 7.67 recurrences from 15–60 dpi (n = 9). Guinea pigs treated with propranolol, the non-specific β-blocker, experienced a 43.55% decrease in cumulative recurrences compared to the infected guinea pigs that were untreated (*p* = 0.018, n = 9). The group treated with a combination of atipamezole and propranolol also experienced a significant reduction in cumulative recurrences, compared to the untreated, infected guinea pigs (39.12% reduction, *p* = 0.021, n = 9). Guinea pigs treated with only atipamezole, the α2-AR blocker, had a 29.26% reduction in cumulative recurrences compared to the untreated guinea pigs (*p* = 0.123, n = 9), although this did not reach statistical significance, likely due to animal variability.

### 3.2. Viral DNA Shedding

**Figure 3:**
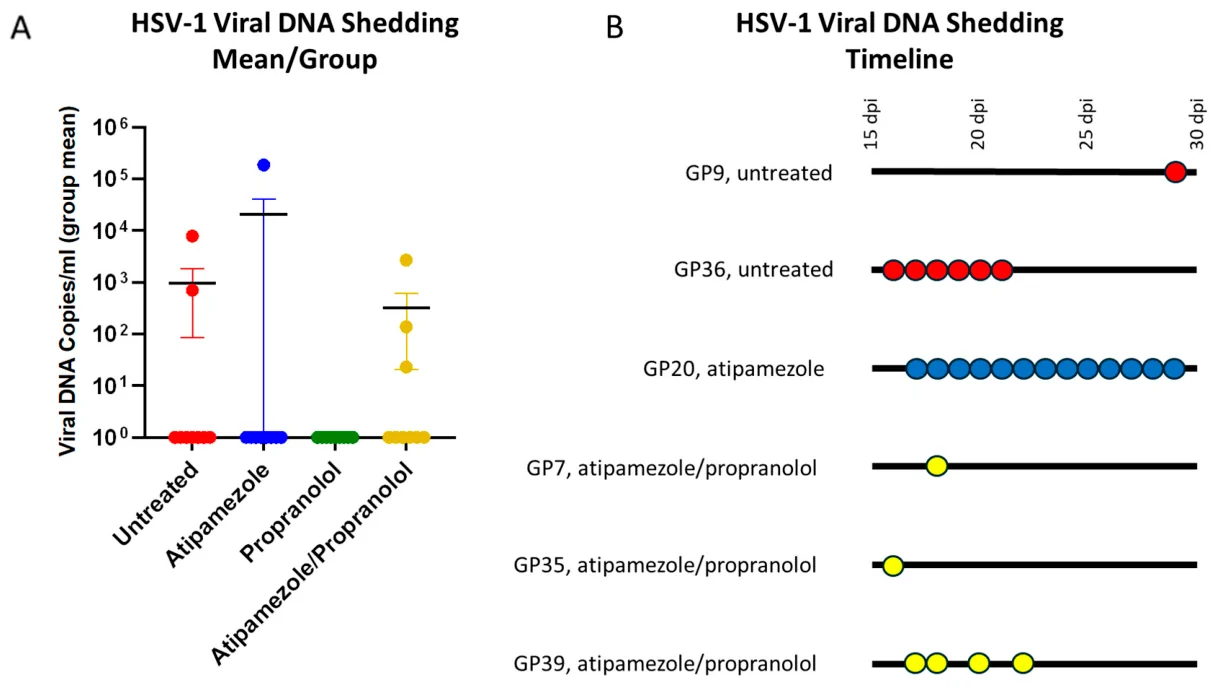
Guinea pig vaginal shedding of HSV-1 DNA. Guinea pigs were vaginally swabbed daily from 15–60 dpi and swabs were analyzed by qPCR for HSV-1 viral DNA. Viral DNA was detected from 6 of 36 total infected guinea pigs (16.67%, n = 9 per group) from 15–29 dpi, with no detectable viral DNA after that time point. (**A**) Vaginal HSV-1 viral DNA quantity. Untreated (n = 9), atipamezole (n = 9), propranolol (n = 9), atipamezole/propranolol (n = 9). No significant differences were found in viral DNA copies shed between treatment groups (one-way ANOVA with Tukey’s post hoc test). No HSV-1 DNA was detected in uninfected guinea pigs and these were not included in the figure (n = 6). (**B**) Timeline of shedding. In the untreated group, two guinea pigs (GP9 and GP36) shed detectable viral DNA for a total of 7 days, indicated by dots on the bar in the figure. One guinea pig in the atipamezole group (GP20) shed HSV-1 viral DNA vaginally for a total of 13 days. No viral DNA was detected from swabs from guinea pigs treated with propranolol. In the atipamezole/propranolol group, three guinea pigs (GP7, GP35, GP39) shed viral DNA for a total of 6 days. No HSV-1 viral DNA was detected from swabs from uninfected guinea pigs.

To determine if the AR inhibitor treatments reduced viral DNA shedding, vaginal swabs were collected daily for all guinea pigs remaining in the study from 15–60 dpi (n = 36 total infected guinea pigs and n = 6 uninfected guinea pigs). Viral DNA in the swabs was quantified by qPCR (Figure 3A). Viral DNA shedding was detectable in 16.67% of guinea pigs (six of 36 total infected guinea pigs) between 15–29 dpi, with no HSV-1 DNA detected in swabs after 29 dpi. In the untreated group, two of the nine guinea pigs shed detectable HSV-1 DNA, with one guinea pig (GP9) having one episode of shedding (21,080 copies) and the other (GP36) shedding for 6 consecutive days from 16–21 dpi (totaling 23,706,162 viral DNA copies), resulting in an average of 956.09 copies of HSV-1 DNA/guinea pig/day (Figure 3A). One of the nine guinea pigs (GP20) treated with atipamezole shed detectable virus for 13 consecutive days, leading to a group average of 20,785 copies of HSV-1 DNA (Figure 3A) (Figure 3B). No other atipamezole-treated guinea pigs shed detectable viral DNA. For the duration of swab collection (15–60 dpi), the propranolol-treated group did not shed any detectable HSV-1 DNA (n = 9). The atipamezole/propranolol treated group shed an average of 318 copies of HSV-1 DNA (Figure 3A), with three of the nine guinea pigs shedding low quantities of detectable HSV-1 DNA for 6 of the 29 days; GP7 and GP35 each experienced a single day of viral DNA shedding and GP39 had 4 days of detectable viral DNA shedding (Figure 3B). Uninfected guinea pigs had no detectable HSV-1 viral DNA from swabs. No significant differences were found between treatment groups in the quantity of viral DNA copies shed or the number of days shedding.

### 3.3. HSV-1 DNA Load and Viral Gene Expression in Nervous System Tissues

To determine if the drug treatments altered the latent viral load in nervous system tissues, we quantified HSV-1 viral DNA in sensory, sympathetic, and parasympathetic tissues that innervate the genital tract. The lumbo-sacral dorsal root ganglia (DRG), the sensory ganglia that innervate the genitals, are the classical site of latency following genital infection. Sensory neurons in the DRG have bifurcated axons, with one branch extending to the periphery to collect sensory information and the other branch extending to the spinal cord to send sensory information to the central nervous system (CNS) via the dorsal horn of the spinal cord [36,37]. The autonomic nervous system includes sympathetic and parasympathetic divisions. Presynaptic (preganglionic) sympathetic neurons are located in the thoracic and lumbar regions of the spinal cord (T1–L3), projecting axons to synapse on postganglionic neurons in the sympathetic chain, which is close to and runs parallel to the spinal cord, fusing into the ganglion impar or sacral sympathetic ganglion (SSG) at the posterior terminal of the sympathetic chain. Postganglionic neurons in the distal sympathetic chain, including the SSG, extend projections to their target organs, which include the vagina, vulva, rectum, and urethra [38]. The second division of the autonomic nervous system is the parasympathetic nervous system, which is generally regarded for its “rest and digest” functions. Presynaptic parasympathetic neurons are located in the sacral spinal cord (S2-S4), extending axons through pelvic splanchnic nerves to the major pelvic ganglia (MPG). Postsynaptic parasympathetic neurons then innervate the pelvic organs, including the genitals, bladder, and rectum [39].

After the study ended at 60 dpi, tissues were collected from guinea pigs in each infected group when no recurrent lesions were present, for quantification of HSV-1 latent viral DNA. Tissues were also collected from three uninfected guinea pigs. Sensory dorsal root ganglia (DRG, n = 8/group), sacral sympathetic ganglia (SSG, n = 7/group), and lumbar spinal cord (L-SC, n = 7/group) were analyzed to quantify latent viral DNA in the sensory and sympathetic neurons that innervate pelvic organs and skin, using a qPCR assay specific for the viral thymidine kinase gene (TK). Additional tissues were collected into paraformaldehyde from infected (DRG, n = 1/group; SSG, n = 2/group; and L-SC, n = 2/group) and uninfected (n = 3 for each tissue) guinea pigs.

**Figure 4:**
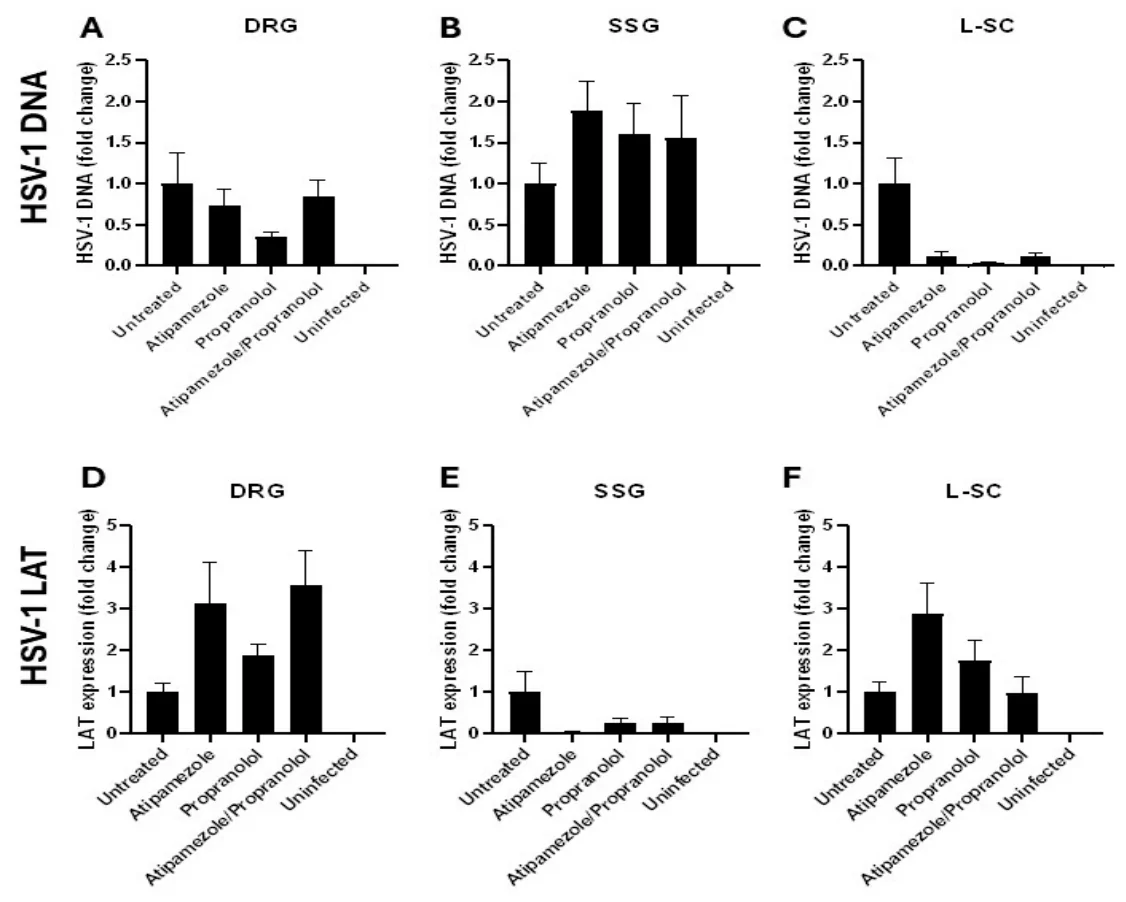
HSV-1 viral DNA load and LAT expression during latency. Sensory dorsal root ganglia (DRG, n = 8/infected group + 3 uninfected controls), sacral sympathetic ganglia (SSG, n = 7/infected group + 3 uninfected controls) and the lumbar region of the spinal cord (L-SC, n = 7/group + 3 uninfected controls) that contains the pre-ganglionic sympathetic neurons were collected from guinea pigs when no lesions were present. HSV-1 viral DNA was quantified by qPCR and HSV-1 LAT expression was quantified by RT-qPCR. HSV-1 DNA in DRG (**A**), SSG (**B**), L-SC (**C**). LAT expression in DRG (**D**), SSG (**E**), and L-SC (**F**). No statistically significant differences were found between treatment groups and the untreated group (one-way ANOVA with a Dunnett’s post hoc test).

Compared with the HSV-1-infected but otherwise untreated guinea pigs, we detected a 1.36-fold decrease in HSV-1 DNA in the DRG of the group treated with atipamezole, a 2.78-fold decrease in the propranolol-treated group, and a 1.18-fold decrease in the atipamezole/propranolol treated group (Figure 4A). In the sacral sympathetic ganglia (SSG), where post-ganglionic sympathetic neurons that innervate the genitals are located, we detected a 1.89-fold increase in the amount of viral DNA present in the atipamezole group, a 1.61-fold increase in the propranolol group, and a 1.55-fold increase in the atipamezole/propranolol group compared to the untreated group (Figure 4B). In the lumbar region of the spinal cord (L-SC), where preganglionic sympathetic neurons are located, the quantity of viral DNA was decreased between 8.47-fold (atipamezole and atipamezole/propranolol-treated groups) and 33.33-fold (propranolol-treated group) compared to the untreated group (Figure 4C). However, none of these differences in DRG or preganglionic and postganglionic sympathetic tissues reached statistical significance.

The DRG, SSG, and L-SC from guinea pigs with no visible lesions were also assayed for expression of the latency-associated transcript (LAT) by two step RT-qPCR. We identified an inverse relationship between LAT expression and quantity of viral DNA. In the DRG, LAT expression in the treated groups was 1.9- to 3.58-fold greater than the untreated group (Figure 4D). In the SSG, LAT expression in the treated groups was reduced 3.7-fold to 26.3-fold compared to untreated guinea pigs (Figure 4E). In the lumbar spinal cord (L-SC), LAT expression was increased in the groups treated with atipamezole (2.9-fold increase) and propranolol (1.76-fold increase) compared to the untreated group, while the combined treatment with atipamezole and propranolol was similar to the untreated group (1.03-fold decrease) (Figure 4F). No statistical significance was found between treated groups and the untreated group, as analyzed by one-way ANOVA with a Dunnett’s post hoc test.

To assess changes in viral DNA in sensory and sympathetic tissues during recurrences, we also collected neuronal tissues from guinea pigs in the untreated group (n = 5) and in each treatment group (n = 6/group) with active recurrent lesions at the time of euthanasia, as well as three uninfected controls. Additional tissues were collected into paraformaldehyde from infected guinea pigs (n = 1/group).

**Figure 5:**
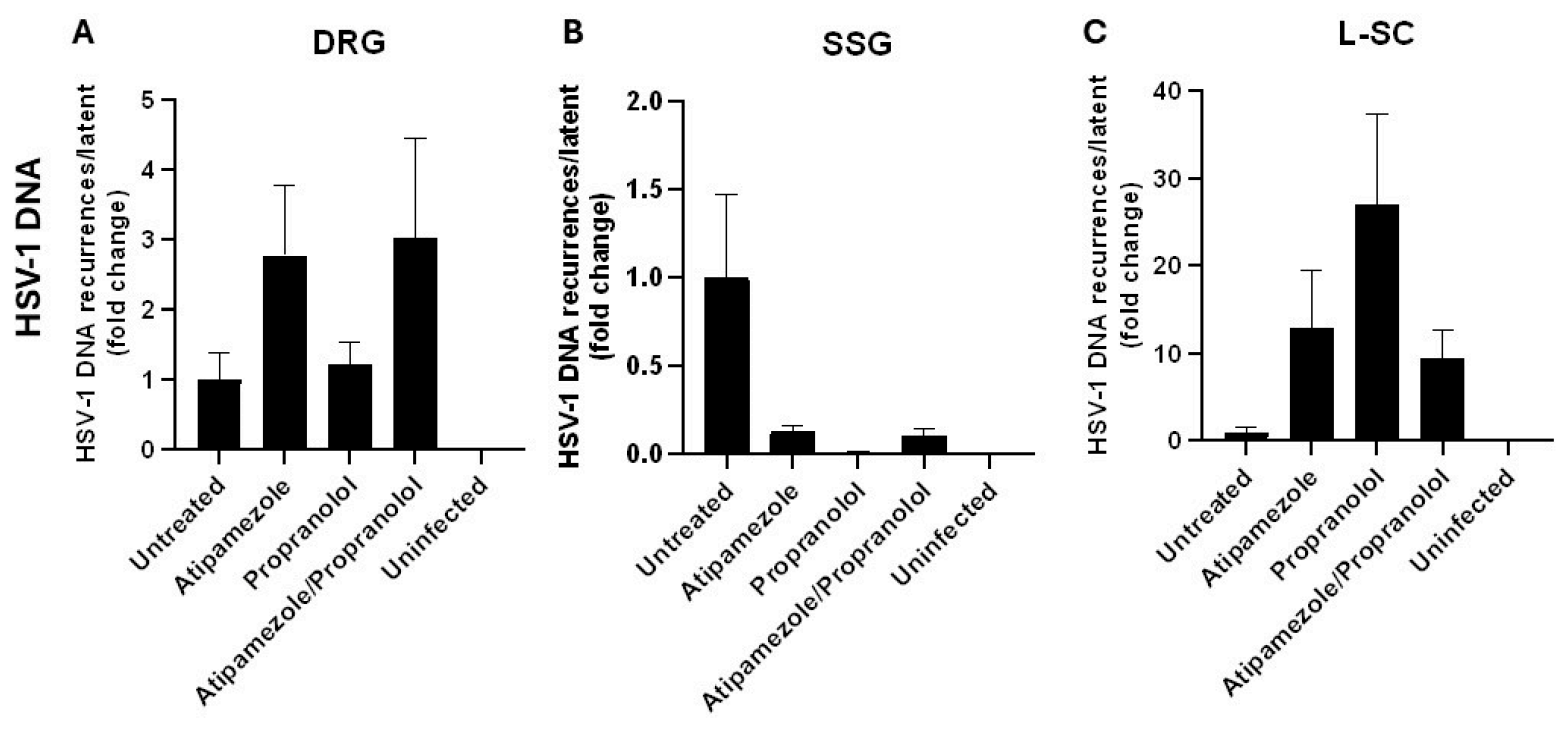
HSV-1 DNA load during recurrences. Dorsal root ganglia (DRG), sacral sympathetic ganglia (SSG) and lumbar spinal cord (L-SC) were collected from guinea pigs when they had a visible lesion (untreated, n = 5; atipamezole, n = 6; propranolol, n = 6; atipamezole/propranolol, n = 6; uninfected, n = 3). HSV-1 DNA in DRG (**A**), SSG (**B**), L-SC (**C**). Data are shown as viral DNA during recurrences/viral DNA during latency, compared as fold change to untreated guinea pigs. No statistical significance was found (one-way ANOVA with a Dunnett’s post hoc test).

Neuronal tissues were processed in the same manner as the guinea pig neuronal tissues from animals with no visible lesions. Viral DNA was quantified by qPCR and normalized to average latent viral DNA load (recurrences/latent, for each respective group) and shown as fold change compared to untreated guinea pigs. In guinea pigs with an active recurrence at the time of euthanasia, viral DNA quantity increased in the DRG 2.77-fold in the group treated with atipamezole, compared to the untreated group (Figure 5A). HSV-1 DNA increased 1.20-fold in the propranolol group and 3.02-fold in the atipamezole/propranolol group (Figure 5A), showing a modest increase in viral DNA in the DRG of treated animals. In the SSG, the viral DNA for guinea pigs with recurrences was decreased by 8.13-fold in the atipamezole group, 102.438-fold in the propranolol group, and 9.80-fold in the atipamezole/propranolol group, compared to the infected untreated group (Figure 5B), suggesting that reactivation and viral replication did not efficiently occur in the SSG of the guinea pigs that were treated with the alpha and beta blockers. In the L-SC, HSV-1 DNA increased by 12.99-fold in the atipamezole group, by 27.04-fold in the propranolol group, and by 9.51-fold in the atipamezole/propranolol group when compared to the infected untreated guinea pigs, showing increased viral DNA in the lumbar spinal cord of treated animals, compared to the untreated guinea pigs (Figure 5C).

## 4. Discussion

Studies in humans, animal models, and cell culture support a strong correlation between increased stress and reactivation of HSV-1. In humans, HSV-1 reactivation is correlated with long-term stress, associated with increased cortisol levels, and acute psychological stress, likely caused by activation of the hypothalamic–pituitary–adrenal (HPA) axis and release of epinephrine [15,16,40,41,42]. We previously showed that epinephrine induced HSV-1 reactivation in cultured primary adult sympathetic neurons, but not in sensory or parasympathetic neurons [10]. Reactivation only occurred when at least two adrenergic receptors (ARs) were activated, which included any combination of α2, β1, and β2 ARs [10]. We hypothesized that blocking these adrenergic receptors after HSV-1 infection would result in a reduction in clinical recurrences in the guinea pig genital model of infection.

Our findings indicate that atipamezole (α2 blocker) and propranolol (β blocker) reduced clinical recurrences. Although the drugs did not have a significant effect on the quantity of viral DNA or expression of LAT during latency, we observed changes in viral DNA quantity during recurrences. In sensory DRG, viral DNA was largely unaffected during recurrences but was substantially less in sympathetic sacral ganglia (SSG) in guinea pigs treated with AR inhibitors compared to untreated guinea pigs. Specifically, propranolol-treated guinea pigs had 100-fold less viral DNA in sympathetic SSG during recurrences compared to untreated guinea pigs. In animals treated with atipamezole or both atipamezole and propranolol, viral DNA in the SSG was 8–10-fold less during recurrences. These results suggest that the overall reduction in clinical recurrences resulted from blocking the effects of epinephrine on peripheral sympathetic neurons innervating the genitals, preventing reactivation in sympathetic SSG neurons while still allowing the virus to reactivate and replicate in sensory DRG neurons.

When a threat is perceived, a rapid physiological response is triggered by the sympathetic-adreno-medullar (SAM) system, activating the HPA axis, leading to increased secretion of epinephrine and norepinephrine from the adrenal medulla into circulation [25,43]. The released epinephrine binds to adrenergic receptors (ARs), which are G protein-coupled receptors (GPCRs) found on numerous cell types throughout the body, including sensory and sympathetic neurons [18]. Several types of adrenergic receptors exist, including α1, α2, β1, β2, and β3, all of which are associated with specific G protein alpha subunits. In the nervous system, α2-, β1-, and β2-ARs are strongly expressed in peripheral sensory and autonomic neurons that innervate the genitalia [18]. β3-ARs are primarily expressed in adipose tissue, and we previously determined that they are not expressed on neurons of interest in pelvic innervation [18]. When the AR is bound by epinephrine, the G protein alpha (α) subunit with which the receptor is associated separates from the beta/gamma (β/γ) complex, initiating intracellular signaling cascades that activate a cellular response. Several different Gα subtypes exist, varying depending on the G protein receptor and its associated signaling pathway. The α2-ARs in association with the Gαi/o subunit inhibit adenylate cyclase, decreasing the amount of cyclic adenosine monophosphate (cAMP) within the cell, which in turn inhibits protein kinase A (PKA). However, the α2-ARs can also activate Src family kinases (35). These α2-ARs are widely expressed in the nervous system, as well as liver, kidney, and spleen. β1-ARs, associated with stimulatory Gαs subunits, activate adenylate cyclase to increase cAMP, primarily in cardiac cells and neurons (35), while β2-ARs can interact with either stimulatory Gαs or inhibitory Gαi/o to modulate activation of adenylate cyclase in bronchial smooth muscle, skeletal muscle, blood vessels, and the nervous system [43]. When the Gα subunit dissociates from the β/γ subunits, the β/γ complex also acts on various effectors, including G protein-coupled inwardly rectifying potassium channels (GIRKs) [44], phospholipase C (PLC) [45], phosphoinositide 3-kinase (PI3K) [46], Src family kinases [47], and G protein-coupled kinases (GRKs) [48,49,50,51]. Thus, epinephrine binding to its various receptors can activate or inhibit multiple signaling pathways that may act on HSV within neurons [18].

Atipamezole is a highly selective and potent α2-AR antagonist that competitively blocks epinephrine from binding the receptor, inhibiting downstream signaling events. Since the α2-AR is coupled to the inhibitory Gα subunit Gαi/o, which inhibits adenylate cyclase, blocking this receptor leads to decreased intracellular cAMP and lower protein kinase A (PKA) activity. Blocking the receptor also prevents dissociation of the β/γ complex from the α subunit, which prevents opening of potassium channels and other downstream effects [52,53]. The α2-ARs in the spinal cord also inhibit nociceptive neurons centrally and reduce substance P release, causing anesthetic effects [54], which are countered by atipamezole to reverse sedation from α2-AR agonists in veterinary medicine [55]. Atipamezole is rapidly distributed to target tissues, producing its major effects centrally, rather than in peripheral tissues [56], which may explain the reduced effectiveness of this particular AR inhibitor [56]. In our studies, treatment with atipamezole reduced HSV-1 recurrences by 29%, which one could argue is a biologically significant reduction, although statistical significance was not achieved. During recurrences, viral DNA load was slightly increased (~3-fold) in sensory ganglia (DRG) compared to untreated guinea pigs but was slightly reduced (~8–10-fold) in the peripheral sympathetic ganglia (SSG), suggesting dichotomous pharmacological effects of atipamezole on sensory and sympathetic neurons. Interestingly, atipamezole treatment increased LAT expression approximately 3-fold in DRG and lumbar spinal cord compared to untreated guinea pigs. Although the increased LAT expression did not reach statistical significance, higher levels of LAT expression contribute to the establishment and maintenance of latency and enhance the capacity for reactivation [57,58]. LAT was previously shown to be responsive to adrenergic stimulation through cAMP and deletion of a cAMP response element in the LAT promoter impairs HSV-1 reactivation [59,60]. However, the specific mechanism by which LAT and epinephrine-induced signaling pathways interact to regulate latency and reactivation has not been determined.

Propranolol is a non-specific β-adrenergic receptor blocker that prevents binding of epinephrine to β1 and β2 adrenergic receptors, preventing activation of the associated G proteins and downstream signaling cascades. While β1-ARs couple with the stimulatory Gαs, activating the cAMP and PKA pathway, β2-ARs can switch between the Gαs subunit and the inhibitory Gαi/o subunit, either activating or inhibiting the cAMP pathway, as well as the Src signaling cascade [61,62]. Since our previous studies showed that at least two ARs, including any combination of α2-, β1-, and β2-ARs, must be activated by epinephrine simultaneously to induce HSV-1 reactivation [10], modulation of the cAMP pathway, and potentially the Src pathway, appears to be essential for optimal HSV-1 reactivation. Activation of these ARs also results in dissociation of the G protein β/γ complex from the α subunit, raising the possibility that the β/γ complex and its downstream signaling pathways play a role in maintenance and reactivation of HSV-1 latency. Binding of epinephrine to β-ARs has also been shown to enhance glucocorticoid receptor transactivation in hippocampal neuronal cells via the β/γ complex stimulation of PI3-K, which may modulate the stress response through an interaction between adrenergic and glucocorticoid receptors in sympathetic neurons [63]. Previous studies have also shown that glucocorticoids, including corticosterone and dexamethasone, can directly reactivate HSV-1, HSV-2, and bovine herpesvirus (BoHV) in primary neuronal cell cultures and in vivo in mice and calves [10,13,64,65]. Sympathetic neurons express both adrenergic and glucocorticoid receptors [18,66]. While both adrenergic and glucocorticoid hormones can independently reactivate HSV, these stress hormones may also interact to enhance reactivation during a stress response.

Previous animal studies have used epinephrine to induce HSV-1 reactivation in both rabbit and mouse ocular models of infection, employing iontophoresis to drive the epinephrine into tissues [35,67,68,69,70,71,72,73,74,75,76,77,78,79,80]. Although epinephrine has a greater effect on sympathetic neurons compared to sensory neurons, the electrical stimulation used to drive epinephrine through the surface epithelium during iontophoresis could potentially stimulate sensory nerve terminals more intensely, resulting in reactivation from either sensory trigeminal (TG) or sympathetic superior cervical (SCG) ganglia [81]. In previous studies, infectious HSV-1 was recovered from the TG and SCG [76], as well as the ophthalmic branch of the trigeminal nerve and the trigeminal root entry zone [72,78], after use of epinephrine iontophoresis. In some cases, 0.1–1.0% 6-hydroxydopamine has been used in combination with epinephrine in these animal models [69,71,73,74,79]. As a synthetic monoaminergic neurotoxin, the dose of 6-hydroxydopamine used may have been sufficient to cause temporary chemically induced nerve damage, resulting in hypersensitivity to epinephrine in sympathetic nerve terminals, which could also affect HSV-1 reactivation efficiency [82]. The reproducible reactivation, peripheral lesions, and viral shedding episodes in mice and rabbits following epinephrine treatment support the significant involvement of epinephrine and the sympathetic nervous system in HSV-1 reactivation [77,79]. Since the guinea pig infection model relies on spontaneous recurrences, our approach used AR inhibitors in an attempt to reduce recurrences caused by epinephrine without the need for exogenous reactivation stimuli.

Previous studies have also attempted to use adrenergic inhibitors in the rabbit and mouse ocular models to assess their effects on HSV-1 recurrences and viral shedding, with mixed results. Alpha-AR inhibitors, thymoxamine and corynanthine, had little to no significant effect on HSV-1 shedding following ocular infection in rabbits [74] or ocular shedding and latent virus from TGs in mice [83]. We observed similar results in the guinea pig genital model, with atipamezole or a combination of atipamezole and propranolol having minimal effects on overall viral DNA shedding quantity or frequency, even though atipamezole reduced clinical recurrences. The β-AR inhibitors, propranolol and timolol, decreased HSV-1 corneal lesions and viral shedding in the rabbit ocular model in previous studies, using epinephrine and hyperthermia as reactivation stimuli [80,84]. In our guinea pig genital studies, which rely on spontaneous reactivation rather than an induction stimulus, we found a 43.5% reduction in genital recurrences and no detectable shedding of viral DNA in guinea pigs treated with propranolol. Although we found no statistically significant differences in vaginal shedding of viral DNA among treatment groups, likely due to animal variability, propranolol does appear to contribute to reducing recurrences and limiting viral shedding, in both the rabbit ocular model and the guinea pig genital model of HSV-1 infection.

After inducing reactivation with epinephrine iontophoresis in previous studies using an ocular model of infection, HSV-1 DNA was found in sensory TG and sympathetic SCG neurons [72,76,78]. Additionally, HSV-1 DNA was found to be decreased in the TG in mice given propranolol two days prior to ocular reactivation induced by epinephrine iontophoresis [85]. This study, however, did not analyze the sympathetic SCG so the relative effects on sensory compared to sympathetic neurons were not assessed [85]. We analyzed viral DNA in sensory and sympathetic ganglia innervating the genitals to identify which neurons were responsible for recurrences when blocking the adrenergic receptors. Following genital infection in guinea pigs, HSV establishes latency in sensory DRG and sympathetic SSG, and substantial quantities of viral DNA are also found in the lumbosacral spinal cord of infected guinea pigs. During recurrences in vaginally infected guinea pigs, viral DNA quantities increase in sensory DRG, sympathetic SSG, and lumbosacral spinal cord [30,31]. Treatment with the AR inhibitors atipamezole, propranolol, or both atipamezole and propranolol had modest effects on the quantity of HSV-1 viral DNA in the sensory ganglia. However, these guinea pigs had less viral DNA in the sympathetic ganglia compared to untreated guinea pigs during recurrences, suggesting that the inhibitors primarily acted on sympathetic ganglia to reduce recurrences. Based on the relative increases in HSV-1 viral DNA in sensory and sympathetic ganglia during recurrences, reactivation still occurred in the sensory ganglia, but not the sympathetic ganglia, leading to the reduction in total clinical recurrences but not complete prevention.

In humans, HSV-1 has been reported to infect the spinal cord and can reactivate causing neurological disease, such as acute transverse myelitis [86]. After HSV-1 genital infection in guinea pigs, we showed that AR inhibitors led to a 10–27-fold greater increase in viral DNA in the lumbosacral spinal cord during recurrences compared to untreated guinea pigs. The consequences of increased HSV-1 DNA in the spinal cord during recurrences are unclear, since no clinical signs were observed in the guinea pigs beyond peripheral lesions. Preganglionic sympathetic neurons are resident in the lumbar cord, but sensory neuron synapses, interneurons, and motor neurons are found in the spinal cord as well. The specific cells in the guinea pig spinal cord that support HSV-1 replication have not been identified but HSV-1 has been detected in the spinal cord of multiple animal models [29,31,87,88,89,90,91,92,93,94]. Viral spread to the spinal cord was found to be independent of the sensory DRG in guinea pigs [29], but in rats, HSV-1 spreads through small cutaneous afferents to laminae I-II and through sympathetic nerve fibers to preganglionic neurons [90]. In our study, it is likely that this large increase in DNA in the spinal cord is from a neuronal type other than sympathetic neurons, since we used AR inhibitors that would have prevented excitation of these neurons. Alternatively, the inhibitors may not have effectively reached the spinal cord sympathetic neurons, allowing viral replication within the central sympathetic neurons while replication was inhibited in the peripheral sympathetic neurons. This hypothesis is supported by the substantial increase in viral DNA in the lumbosacral spinal cord in propranolol-treated guinea pigs, as opposed to the modest increase in atipamezole-treated guinea pigs, since atipamezole rapidly crosses into the central nervous system within minutes, as opposed to propranolol, which can take up to four hours to reach maximum concentrations in the spinal cord [95].

## 5. Conclusions

We have shown that AR inhibitors atipamezole and propranolol, or a combination of the two, reduced the cumulative number of genital HSV-1 recurrences in the guinea pig genital model of infection. AR inhibitors prevent epinephrine from binding to and activating the G proteins coupled to the receptors. Since the G protein α subunits can either activate or inactivate cAMP and Src signaling pathways, we speculate that both stimulatory and inhibitory G protein α subunits, as well as the β/γ complex, are involved in a complex interplay to regulate the maintenance of latency and reactivation of HSV-1 in sympathetic neurons. Increased cAMP has been shown to induce reactivation in neuroblastoma cells [96] and may be required for HSV-1 reactivation in sympathetic neurons. Both cAMP and Src signaling pathways can regulate the binding of the cAMP response element binding protein (CREB) to the HSV-1 genome [10,60]. The latency-associated transcript (LAT) also plays a role in regulating latency and reactivation, and deleting a cAMP response element in the LAT promoter reduces epinephrine-induced reactivation in the rabbit eye model [67,70,75,97,98]. Further studies are needed to define the precise relationship between these interacting elements, how regulation differs in sensory and sympathetic neurons, and the molecular mechanisms by which AR inhibitors, such as propranolol, alter these interactions. Propranolol is a widely prescribed drug used to treat a variety of health concerns. This study provides preclinical evidence suggesting propranolol and other beta-AR inhibitors may be useful in reducing HSV-1 genital recurrences originating from sympathetic neurons.

## Data Availability

Data are available upon request.

## Abbreviations

The following abbreviations are used in this manuscript:

HSV-1	Herpes simplex virus 1
DRG	Dorsal root ganglia
SSG	Sacral sympathetic ganglia
MPG	Major pelvic ganglia
L-SC	Lumbosacral spinal cord
